# Human-Leopard Conflict: An Emerging Issue of North China Leopard Conservation in Tieqiaoshan Provincial Nature Reserve in Shanxi Province, China

**DOI:** 10.3390/ani10060996

**Published:** 2020-06-07

**Authors:** Kahindo Tulizo Consolee, Chunyv Gao, Kasereka Vitekere, Chunshi Li, Hua Yan, Guangshun Jiang

**Affiliations:** 1 College of Wildlife and Natural Protected Areas, Northeast Forestry University, Harbin 150040, China; tulizok@yahoo.com (K.T.C.); cgy46829472567@163.com (C.G.); kasvitekere@gmail.com (K.V.); lcs981224@163.com (C.L.); 2 Guangdong Provincial Key Laboratory of Silviculture, Protection and Utilization, Guangdong Academy of Forestry, Guangzhou 510520, China

**Keywords:** livestock depredation, north China leopard, reserve, seasonal, local community

## Abstract

**Simple Summary:**

One of the main conservation issues, both locally and globally, is the issue of human-wildlife conflict. Losses of livestock due to predation by carnivores such as leopards has become a common problem. Residents share negative attitudes toward leopards due to conflicts over the depredation of livestock. Using data obtained from League Cat Forest Department records, and standardized, structured and semi-structured questionnaires, we found that the north China leopard was in serious conflict with the locals, causing them personal economic losses. Residents noted that the species was very dangerous to their livestock, causing them economic loss, and wanted to reduce or even eliminate it from their area.

**Abstract:**

Livestock depredation by large carnivores is a conventional human–wildlife conflict, both at the local and regional level. Many species of wildlife have become endangered because of this conflict. In this study, an investigation of livestock depredation was conducted for the north China leopard in and around Tieqiaoshan Provincial Nature Reserve in Shanxi Province between 2015 and 2018. Data were obtained from League Cat Forest Department records. Additionally, standardized, structured, and semi-structured questionnaires were used to collect data with the help of reserve field staff. The results show that there was a significant difference (*p* = 0.015) in livestock depredation in various seasons of the year; the highest depredation was recorded in spring, followed by summer. A significant difference (*p* = 0.02) was observed between cattle and other livestock species, showing that more cattle were killed by the north China leopard. Most of the livestock depredation occurred during late morning and evening, likely because the leopards are crepuscular. Residents in and around the reserve suffered a high economic loss, ranging between RMB 5000 and 10,000 (USD 706.76–1413.53) per year in terms of the estimated market price of the killed livestock. The attitudes of residents towards the north China leopard vary according to the economic activities of the locals, with about 76% of the livestock keepers reporting that the leopard is “very dangerous” and 8% of the arable farmers in and around the reserve indicating that leopard is “very dangerous.” We recommend that a system with local participants would ensure more effective management of human-north China leopard conflict, as it would allow local communities to take greater responsibility.

## 1. Introduction

According to [1], human–wildlife conflict is a wildlife conservation issue defined as any interactions leading to negative impacts on the humans or wildlife involved [2]. Some of the factors attributed to increasing human-wildlife conflicts include land-use change, wildlife habitat fragmentation and loss, increase in the human population, climate change and variability, among many others [3,4]. These factors have changed the resources for wildlife, livestock, and humans, escalating competition for space, forage, and water. Among the forms of human–wildlife conflicts, livestock depredation has been singled out as the common conflict in areas that are close to forest edges and in areas that provide cover for large carnivores to come within reach of livestock unnoticed [5,6].

Leopards are considered among the top predator in their range, where they play a crucial role in the continuation of biodiversity [7,8]. As a result of large home ranges, leopards come into conflict with humans, especially in areas where livestock keeping overlaps with the leopard range [5,7,9]. Leopards often attack livestock that are grazing in and around forest areas [10,11]. Moreover, they pose a risk to human lives inside human settlements [5]. This damage to local livelihoods often angers the livestock owners, who may resort to taking revenge [12]. Since livestock keeping forms sources of income for the local people, predation by leopards angers the local community, enhancing the appetite for retribution among the affected people [13]. The loss of sources of income intensifies the level of anger toward predators, enhancing the appetite for retribution among the affected stockbreeders [13,14].

The north China leopard *P. pardus japonensis*, commonly referred to as *P. pardus fontanierii* in the Chinese literature, is the most widespread subspecies of leopard in China. Recent camera-trap surveys and other evidence revealed the presence of the north China leopard in Shanxi province in and around the protected area of the Tieqiaoshan Provincial Nature Reserve [15]. Similarly to the situation reported by [16] for tigers *Panthera tigris* in India and Nepal, the north China leopard is facing the challenge of lower prey numbers in Tieqiaoshan Provincial Nature Reserve [15]. With the lower numbers of prey, the north China leopard has been reported to prey on livestock in and around Tieqiaoshan Provincial Nature Reserve, negatively impacting the local communities who depend on livestock rearing as the source of income [17,18]. The losses of livestock through predation have led to relationship disconnection between the local people and the wildlife conservation agencies [18]. This conflict has become a significant threat to the conservation of the north China leopard, and the issue has attained the status of national priority due to which the government is under intense pressure from rural communities who require compensation for their livestock preyed on by leopards [15].

This study aims (1) to determine the livestock species which are preyed mostly by the north China leopard and the economic losses incurred by the local communities, (2) to determine the seasonal variation in human–north China leopard conflict in and around Tieqiaoshan Provincial Nature Reserve, and (3) to evaluate the attitudes of local communities in and around the nature reserve toward the conservation of the north China leopard and the livestock depredation compensation schemes. We hypothesized that: (1) livestock species preyed by the north China leopard would vary according to the body size—in particular, sheep will be killed more than cattle because of their small body size; (2) human–north China leopard conflict would show evident seasonal variation, peaking during spring and summer because of actively growing grasses which make the livestock move in and around the reserves; and (3) the attitude of local communities towards the conservation of north China leopard will be negative.

## 2. Materials and Methods

### 2.1. Study Area

**Figure 1:**
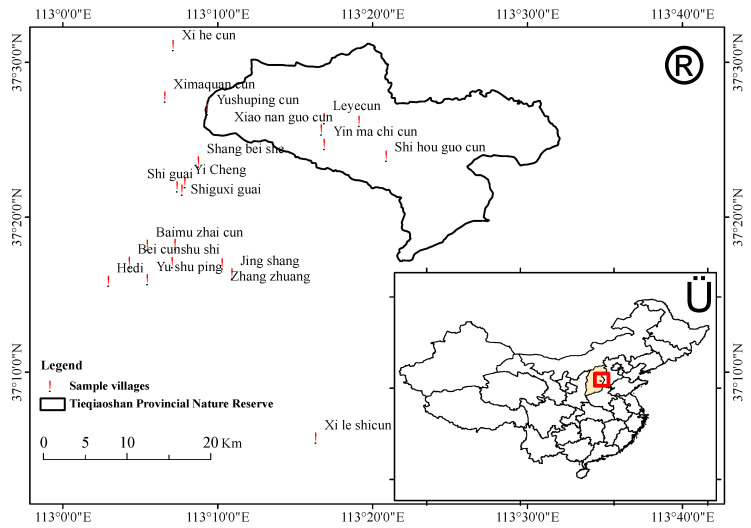
Map showing study area.

This study was conducted in and around Tieqiaoshan Provincial Nature Reserve in Shanxi Province, China (Figure 1). Tieqiaoshan Provincial Nature Reserve was established in 2002, approved and launched by the administration of Shanxi Province People’s Government in 2009. The reserve is located between 113°05′ and 113°35′ E and 37°13′ and 37°34′ N, and the elevation ranges between 1400 m to 1700 m. The total area of the reserve is 35,351.7 ha, with a core area of 13,948.6 ha, and a buffer area of 7401.7 ha. Tieqiaoshan Provincial Nature Reserve is located in an area that is under the influence of warm temperate and continental climate with a little snow in winter, dry wind in spring, strong rainfall in summer and short cooler period in autumn. The average annual rainfall is about 600 mm, which mainly occurs between July and September and the mean annual temperature is about 6 °C. Tieqiaoshan Provincial Nature Reserve is rich in diversity of flora and fauna. One hundred and forty-nine species of wild vertebrates are reported in this reserve, including 3 species of amphibians, 6 species of reptiles, 116 species of birds and 24 species of mammals. The area is characterized by four main types of forest, namely deciduous broad-leaved forest, warm coniferous forest, warm coniferous and broad-leaved mixed forest and deciduous shrubs with high representativeness of *Pinus tabuliformis* forest, *Quercus liaotungensis* forest, *Pinus tabulaeformis*–*Quercus euphratica*, *Betula platyphylla*–*Populus davidiana* forest [19].

### 2.2. Data Collection and Analysis

A part of the data used in this study was obtained from the League Cat Forest Department. The data included incidences of livestock depredation by north China leopards that were available for a 4 year period (2015–2018) and the compensations made by the department on the loss of the livestock. In addition, standardized, structured, and semi-structured questionnaires were administered to 290 local people from 22 villages from the four study areas. Completion of the questionnaires was done ‘face-to-face’ with the respondents, who were randomly selected. To reduce bias, we followed a structured questionnaire, while standardized questionnaires were used to ensure the reliability, generalizability, and validity of the responses. The questionnaire included open-ended questions related to perceptions of conflicts between humans and north China leopard, a set of closed (no/yes/do not know) and socio-demographic variables. In order to collect as much information as necessary, open-ended questions were asked at the end of the structured portion. The heads of households who were identified by the people at the houses, and who were mainly men, were interviewed. However, in their absence, housewives who were willing to participate were interviewed. Most of the livestock keepers in and around the reserve had animal pens within their homestead which were used for short-time confinement of sick cattle, calves or sheep. These animal pens were also used to temporarily hold those livestock, especially sheep, which have just given birth.

The statistical analysis in this study was conducted using SPSS Statistics for Windows Version 17.0. Prior to analysis, the normality of the data was checked using the Kolmogorov-Smirnov Test and the Shapiro-Wilk test. Datasets which were not normally distributed, were ln-transformed to reduce intrinsic variation and minimize the effect of outliers. We excluded outlier predictors with Cook′s distance ~1. One-way ANOVA was used to determine the significant differences among livestock species depredation, time, and seasonal of depredation. The results were presented using the least significant difference (LSD) of groups such as livestock type, time, and season of depredation.

## 3. Results

### 3.1. Attitudes of Local Communities to the North China Leopard and Their Preferred Management Response

The attitudes of local communities to the north China leopard vary according to the local economic activities. A total of 289 local people in and around the nature reserve who were interviewed answered the questions. Out of this cohort, approximately 76% of the livestock keepers reported the north China leopard as being “very dangerous,” and 8% of arable farmers in and around the reserve noted that leopard is “very dangerous”. As for the preferred management measures, the people in and around Tieqiaoshan Provincial Nature Reserve had various opinions. Overall, 80.4% of local people suggested the standardization of production activities of residents living inside and outside the protected areas in order to minimize the interference and threats made by people on leopards, while 78.3% suggested strengthening publicity, environmental education, the management of protected areas, and regulate residents′ production by granting inhabitants some subvention for the development of socio-economic activities. Increasing ecological compensation and minimizing the loss of livestock species, which is a real issue to overcome within these villages, was suggested by 59.8% of the local people. Meanwhile, 68.6% of local people suggested that the government should increase the support for residents in the reserve by promoting land-use zoning to minimize contact between livestock and wildlife, while 31.9% of the local people suggested that the government should diversify sources of income by establishing tertiary industry. Additionally, 66% of livestock keepers suggested that the leopard should be removed from the area and should be installed in another place which is uninhabited by human beings, while 28% of arable farmers recommended that the north China leopard should be removed. About 17.4% of local people suggested that livestock should not be kept within and around the reserve or that the number of livestock should be reduced to manage them as zero-grazing. Introducing other animals as prey in this reserve and enclosing the area where the north China leopard is living was suggested by 9.8% local people.

### 3.2. Incidences of Livestock Depredation and Their Distribution Pattern across the Study Period

**Figure 2:**
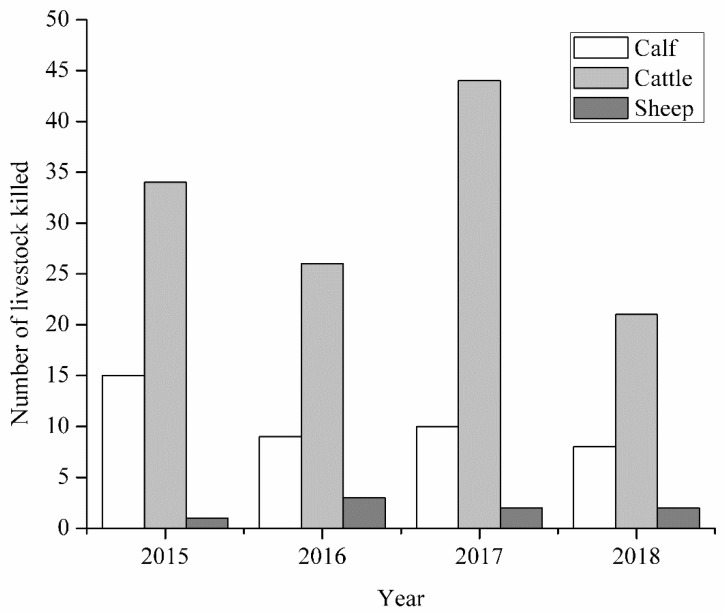
Number of livestock killed by north China leopards in and around the Tieqiaoshan Provincial Nature Reserve.

Figure 2 shows the number of livestock preyed on by the north China leopard within the 4 year study period (2015–2018) in and around the Tieqiaoshan Provincial Nature Reserve. A total of 173 livestock were killed, out of which 125 (72%) were adult cattle, 40 (23%) were calves, and 8 (5%) were sheep. One-way ANOVA revealed that depredation of livestock by the north China leopard differ significantly among the groups (*p* = 0.017). An LSD post hoc test indicated that the number of cattle preyed on was significantly higher than that of calves and sheep. The depredation pattern of calves by the north China leopard depicted a decreased trend from 2015 to 2018 (Figure 2). On the other hand, the depredation patterns of cattle and sheep were irregular. The number of cattle killed in 2017 was significantly higher than in the other years (*p* = 0.001). Further analysis showed that the depredation of livestock occurred at different times of the day. Table 1 provides the daily pattern of livestock killings by north China leopards in and around the reserve. Approximately 20% of the daily livestock killings occurred at dusk, between 5:00 p.m. and 7:00 p.m., while 18.9% of the killings happened in the morning, between 8:00 a.m. and 12:00 noon. About 16.7% of livestock killings occurred at night, between 7:00 p.m. and 12:00 midnight, while 13.3% of daily killings happened in the afternoon, between 12:00 noon and 5:00 p.m. However, 34.4% of killings were reported to occur at an unknown time of the day.

**Figure 3:**
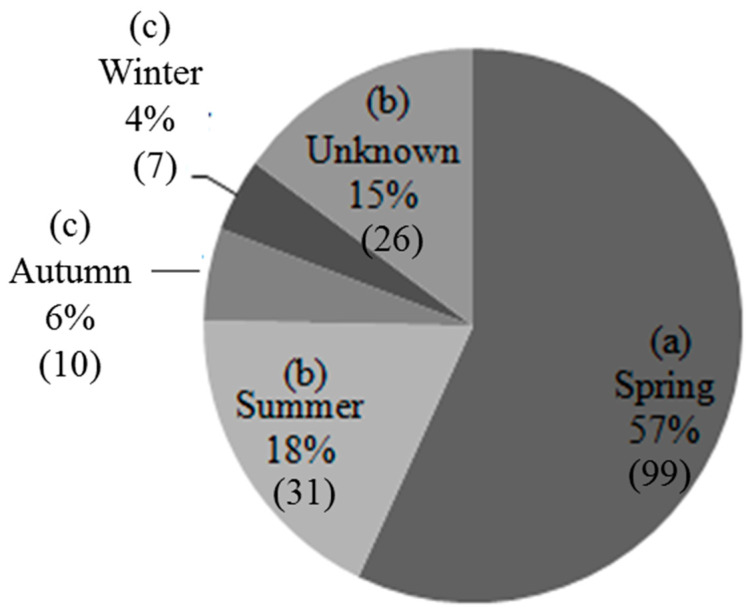
Seasonal incidences of livestock depredation by north China leopards in and around the Tieqiaoshan Provincial Nature Reserve. The percent values with different letters in brackets represent significant differences at *p* = 0.016 level. Numbers in brackets next to % value represent the number of livestock killed in that season.

Seasonally, the highest rate of depredation was recorded in spring months, followed by summer, autumn, and winter (Figure 3). Post hoc tests indicated that approximately 57% of the livestock killings by north China leopards recorded in spring were significantly higher than that of summer (18.3%), autumn (5.4%), and winter (4.3%) (*p* = 0.016). Depredations in summer also differ from those of autumn and winter. An LSD post hoc test revealed a non-significant difference in livestock depredation by the north China leopards between autumn and winter (*p* = 0.109). Note, however, that approximately 15% of the depredations were reported to have happened in unknown seasons (Figure 3).

### 3.3. Sources of Income and Economic Losses Due to Livestock Depredation

The monthly income of the local people living in and around the Tieqiaoshan Provincial Nature Reserve indicated some variations. For approximately 48.5% of people, their monthly income was RMB 1000 (USD 141.32), while 36.7% had monthly income varying between RMB 1000 and 3000 (USD 141.32–423.95). About 6.6% of people earned between RMB 3000 and 5000 (USD 423.95–706.58) per month. People who earned more than RMB 5000 (USD 706.58) per month accounted for 0.5% of the population in and around Tieqiaoshan Provincial Nature Reserve. The primary source of income of local people in and around the Tieqiaoshan Provincial Nature Reserve was cattle keeping, accounting for about 57.1% of total income (Table 2). Other livestock keeping accounted for 4.6% of total income. The income source from arable farming accounted for 28.6% of total income in and around the Tieqiaoshan Provincial Nature Reserve, while 9.7% of income was from other sources (Table 2). There was a high economic loss due to north China leopard depredation in and around this reserve. The loss was estimated according to the market price of killed animals. The mean annual economic loss was estimated to range from RMB 5000 to 10,000 (USD 706.58–1413.17) per person. The League Cat Forestry Department paid total compensation of RMB 209,000 RMB (USD 29,535.21) as compensation for 173 livestock depredations (calf, cow, and sheep), with an average of RMB 1050 RMB (USD 148.38) as compensation per animal.

Although the Government has set up a compensation system for livestock killings by the north China leopard, the perception of this system by the local community is not uniform across all the local communities. Approximately 94.6% of people in and around the reserve think that the amount of compensation from the government is not enough.

## 4. Discussion

Conservation of large carnivores is facing many challenges due to increases in the human population, encroachment of protected areas, unsustainable use of wildlife resources, and growing agricultural needs. People living in and around protected areas are mainly stock owners who earn their livelihood from livestock [20,21]. Large carnivores such as leopards have shown a dietary shift from wild prey species to domestic species [22]. In some regions, such as Pakistan, the leopard has been reported to prey on lizards, rhesus monkeys, snakes, and porcupines. In places where wild prey becomes scarce, the leopard has been reported to attack domestic animals such sheep, goats, cattle, donkeys, and calves [23]. This has caused leopards to be regarded as ruthless by human beings and leopards are considered as a symbol of fear and contempt whenever found [5,24].

This study shows that the number of livestock species killed by north China leopards in and around the Tieqiaoshan Provincial Nature Reserve differed significantly. Among the livestock, north China leopards killed more cattle. This is contrary to the findings of [3,25], who mentioned in their studies that leopard killed more goats, followed by sheep. This could probably be due to the reasons that goats are not domesticated in and around of this reserve, and most of the sheep kept are not free-range. Therefore, the free-range system of the farming of cattle practiced in and around the reserve makes it ideal for the north China leopard to prey them. In the free-range system, the cattle roam freely outdoors rather than being confined in an enclosure for 24 h each day.

Livestock depredation is strongly seasonal, with the highest incidences reported in spring followed by summer. This is likely because, in spring, the ice melts, and the grass grows; hence, the livestock roams around the reserve looking for green pasture. Moreover, it is observed that livestock depredation increases during spring and summer because resident ungulates disperse outside protected areas to pastoral lands where most livestock are found because of actively growing grasses maintained by heavy livestock grazing [2,26,27] observed that a reduced wild prey base and large home ranges could lead to predators turning to livestock. Therefore, during spring and summer, the home range of the residents of ungulates in the reserve increased, leading the north China leopard to prey on the livestock. In North America, the predation of livestock by large carnivores depended on the length of the summer grazing season [28,29]. We found that most of the livestock predation was noted at late morning and evening. This agrees with the study of [30] on patterns of human–carnivore conflict in and around Machiara National Park, Pakistan. North China leopards are crepuscular animals, hence they are active primarily during twilight. This could explain why most of the depredation of livestock happened during late morning and evening. In winter, depredation by north China leopard was low, likely due to heavy snowfall, restricting livestock grazing. We can deduce from this part that strategies for mitigating the depredation of livestock should take seasonality in depredation events into account.

Economic losses incurred by local communities as a result of the loss of their livestock through mass killing by carnivores could contribute to more conflict and poor livelihood. Some studies reported a loss of up to 12% of livestock kept by farmers, which represents half of their average per capita income through depredation [31,32]. In this study, the economic loss due to north China leopard depredation in and around Tieqiaoshan Provincial Nature Reserve was very high. The loss estimated based on the market price of the killed animals was RMB 209,000 (USD 29,544.39). Although the government, through the League Cat Forestry Department, compensates for the animal killings, the attitude by the livestock keepers toward the compensation scheme is negative. More than 90% of the people in and around the reserve reported that the amount of compensation from the government is very low. This shows that the current compensation system does not have the backing of the local people. We suggest that a system with local participants would ensure more effective of management of human-north China leopard conflict, as it would allow local communities to take greater responsibility.

Moreover, the livestock depredation by north China leopards resulted in antagonistic interactions between the residents and the leopard in and around the reserve. The interaction between the leopard and the human is complex, and the relation is dominated by fear [33]. Overall, 66% of local people interviewed reported that the north China leopard is very dangerous, and they suggested that the leopard should be removed from the reserve. The reasons for this negative perception of leopards in and around the reserve could be a combination of (i) recurring financial loss due to livestock killing by leopards, (ii) limited mitigation measures by the government to reduce such incidences, (iii) lack of sustainable and alternative source of livelihood opportunities, (iv) lack of education awareness about leopards and their behavior and (v) probable conflict since historic times. Some of these perceptions were also reported in the studies of [3,5]. The dictum that “nothing operates in a vacuum” is especially applicable to the management of human–wildlife conflicts. Social, cultural, and political forces are continuously reshaping the current world and its environs.

Therefore, strategies designed to address human–wildlife conflict in this reserve will rest largely on the ability of decision-makers and conservation agencies to involve different stakeholder opinions in formulating the policies. In this study, local people living in and around the reserve suggested several strategies to help mitigate conflict with the north China leopard. This strategy includes the following: diversifying sources of income to the local people through building tertiary industries, enhancing public awareness about leopards and their behavior to the local people, and establishing designated areas for grazing in and around the reserve. The livestock owners decried the poor state of their animal pens in their homestead. The livestock owners felt that construction of predator-proof pens should mitigate animal killings. A similar approach was used in other areas where livestock was killed by large predators [31,34]. Moreover, the grazing the livestock in areas that are away from the core habitat of the leopard should reduce the incidences of attack [26]. In addition, insurance plans that take into consideration the value of animals may be adopted to promote the coexistence of human-north China leopard in and around Tieqiaoshan.

## 5. Conclusions

Based on the findings of this study, we conclude that the north China leopard mainly attacks cattle in and around Tieqiaoshan Provincial Nature Reserve, probably because cattle are kept as free ranging, making it ideal for the nocturnal leopard to prey on them. The depredation of livestock in the reserve strongly varies with season, and the highest incidences were reported in spring. Moreover, most of the livestock depredation occurred in late morning and evening, likely because the leopard is crepuscular. Most of the local people in reserve have a negative attitude towards the conservation of the north China leopard. We recommend that mitigation measures should be implemented in and around Tieqiaoshan Provincial Nature Reserve. Local community heads, children, and women in this reserve should regularly undergo education awareness focusing on the biology of the north China leopard and its patterns of attacks. We further recommend that a system with local participants would ensure more effective management of human-north China leopard conflict, as it would allow local communities to take more responsibility.

## Figures and Tables

**Table 1:** The daily pattern of livestock killings by north China leopards in and around the Tieqiaoshan Provincial Nature Reserve.

Time of the Day	Number of Livestock Killed (n)	Percent (%) of Incidences of Livestock Killings
Early morning (5:00 a.m.–8:00 a.m.)	4	2.2
Morning (8:00 a.m.–12:00 noon)	33	18.9
Afternoon (12:00 noon–5:00 p.m.)	23	13.3
Evening (5:00 p.m.–7:00 p.m.)	35	20.0
Night (7:00 p.m.–12:00 midnight)	28	16.7
Late night (midnight–5:00 a.m.)	8	4.4
Unknown	42	24.5

**Table 2:** Source of income of local people in and around Tieqiaoshan Provincial Nature Reserve.

Sources of Income	Percent (%) Contribution to the Total Income
Cattle keeping	57.1
Other livestock keeping	4.6
Arable farming	28.6
Other	9.7
Total	100.0
